# Effects of calcination temperatures on the structure–activity relationship of Ni–La/Al_2_O_3_ catalysts for syngas methanation†

**DOI:** 10.1039/c9ra09674d

**Published:** 2020-01-27

**Authors:** Hongli Wu, Meng Zou, Lisheng Guo, Fengyun Ma, Wenlong Mo, Yuming Yu, Inamullah Mian, Jingmei Liu, Shuangjie Yin, Noritatsu Tsubaki

**Affiliations:** Key Laboratory of Coal Cleaning Conversion and Chemical Engineering Process Xinjiang Uyghur Autonomous Region, College of Chemistry and Chemical Engineering, Xinjiang University Urumqi Xinjiang 830046 China ma_fy@126.com; Department of Applied Chemistry, School of Engineering, University of Toyama Gofuku 3190 Toyama 930-8555 Japan tsubaki@eng.u-toyama.ac.jp

## Abstract

A series of Ni–La/Al_2_O_3_ catalysts for the syngas methanation reaction were prepared by a mechanochemical method and characterized by thermogravimetric analysis (TG-DTA), X-ray fluorescence (XRF), X-ray diffraction (XRD), N_2_ adsorption–desorption, H_2_ temperature-programmed reduction (H_2_-TPR), and X-ray photoelectron spectroscopy (XPS). The calcination temperatures (350–700 °C) had significant impacts on the crystallite sizes and interactions between NiO and Al_2_O_3_. The catalyst calcined at 400 °C (cat-400) showed a 12.1% Ni dispersion degree and the maximum bound state of NiO (54%) through the Gaussian fitting of H_2_-TPR. Cat-400 also achieved the highest CO conversion, CH_4_ selectivity and yield. Cat-400 exhibited good stability and catalytic activity in a lifetime testing of 200 h. The deactivation of cat-400 was mainly caused by carbon deposition according to the data from XRD, TG-DTG and XPS.

## Abstract 2

Calcination temperature affects the existing types of NiO, and the influence of the three NiO types on the catalytic activity of samples is bound type ≫ free type > combined type.

## Graphical abstract

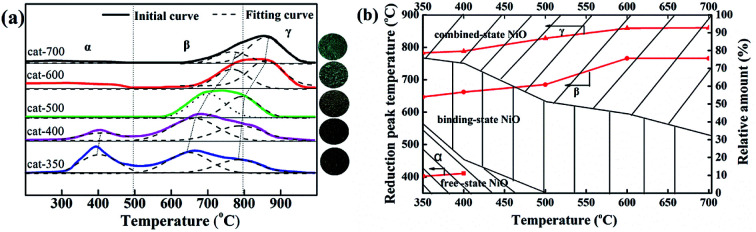

## Introduction

Catalytic methanation has attracted considerable attention since being reported by Sabatier^1^ and has been applied in various industrial processes such as the removal of oxo-compounds (COX) from the feed gas for the synthesis of ammonia,^2^ and in relation to the Fischer–Tropsch synthesis such as producing methane from synthesis gas.^3–7^ The preparation of natural gas from syngas has become an important method for the clean and efficient use of coal. Due to the poor heat transfer and high energy consumption of fixed-bed reactors, a slurry-bed reactor is used for developing the CO methanation reactor to effectively remove the heat and improve the performance. In a slurry-bed reactor, liquid paraffin is used as an inert liquid medium in a strong turbulent state under stirring or gas flow and, therefore, there are little temperature and concentration gradients.^8–10^ There are few reports on CO methanation in slurry-bed reactors.^10–12^

Ni-based catalysts are widely used for CO methanation reactions due to their high activity and a competitive cost compared to noble metals. Nevertheless, despite exhibiting advantageous catalytic properties, there are many factors that affect the catalytic activity.^13–17^ Calcination temperature is an important factor determining the catalytic activity of catalysts.^18–22^Y. Echegoyen *et al.*^18^ investigated the effect of calcination temperature (450, 600, 800 and 1000 °C) on the performance of Ni–Al catalysts for the thermal catalytic decomposition of methane. At the calcination temperature of 600 °C, the Ni–Al catalyst exhibited Ni crystallites of the smallest size (17.9 nm) and the best catalytic activity with a yield of H_2_ at 78%. Numpilai *et al.*^19^ prepared Fe–Co/K–Al_2_O_3_ catalysts for the hydrogenation of CO_2_ to light olefins by a two-step incipient wetness impregnation method. The calcination temperature of Fe–Co/K–Al_2_O_3_ (400–800 °C) significantly impacted the size of metal oxide crystallites, the interaction between Fe_2_O_3_ and other metal oxides, and the transformation of potassium phases, which further affected CO_2_ conversion, product selectivity and the yield of olefins. Dorner *et al.*^20^ investigated the effect of calcination temperature on Fe–Mn–K–Ce catalysts for CO_2_ hydrogenation to olefination. Increasing the calcination temperature from 500 to 800 °C caused a drastic reduction in the specific surface area from 200 to 100 m^2^ g^−1^. Compared with the catalyst calcined at 400 °C, the catalyst calcined at 800 °C exhibited a 5% and 2.9% increase in olefin/paraffin (O/P) ratio and CO_2_ conversion, respectively.

The materials prepared by the mechanochemical method can be regarded as homogeneous materials due to the highly dispersed components, the concentrated distribution of pore size and the specific surface area of over 100 m^2^ g^−1^. These materials exhibited excellent catalytic performances, such as high activity, good selectivity, and long lifetimes.^19–22^ Therefore, the mechanochemical method was gradually applied in the preparation of catalytic materials. Using the mechanochemical method, Xing *et al.*^21^ prepared a visible light photocatalyst, N-doped nano-TiO_2_, which can degrade organic matter in aqueous solution. Květa *et al.*^23^ prepared a Ni/Mo catalyst loaded on Al/Ce composite oxides and applied it in the hydrodesulfurization of 1-benzothiophene. Due to the promising applications of the mechanochemical method, it is necessary to study the effect of calcination temperature on the structure and catalytic performance of Ni-based catalysts for CO methanation in a slurry-bed reactor. In this work, a series of Ni–La/Al_2_O_3_ catalysts were prepared by the mechanochemical method for CO methanation, and the influence of calcination temperature (350–700 °C) on the structure–activity relationship of the Ni–La/Al_2_O_3_ catalyst for the syngas methanation reaction in a slurry-bed reactor was investigated in detail.

## Experimental

### Material synthesis

A ND7-2L planetary ball mill was used as the mechanical chemical reactor, and the grinding ball diameter was 6 mm with a ball/material quality ratio of 2 : 1. The reactants, 15 mmol Ni(NO_3_)_2_·6H_2_O, 98 mmol Al(NO_3_)_3_·9H_2_O, 160 mmol (NH_4_)_2_CO_3_, and 1.3 mmol La(NO_3_)_3_·6H_2_O, were placed into the grinding jar and milled for 1 h with positive and negative alternating rotation. The mixture was dried at 100 °C for 10 h to obtain the precursor, which was further calcined in air for 4 h at 350, 400, 500, 600 and 700 °C, respectively. The calcined samples were screened to 80–120 mesh and placed in a fixed-bed reactor for reduction for 6 h at 850 °C with a flow of H_2_ at 40 mL min^−1^ to acquire the reduced Ni–La/Al_2_O_3_ catalysts. The reduced samples were screened to 60–80 mesh for evaluation. The chemical reactions that occurred in the process of the preparation of the catalyst are as follows:(NH_4_)2CO_3_ + Ni(NO_3_)_2_·6H_2_O = NiCO_3_ + 6H_2_O + 2NH_4_NO_3_3(NH_4_)_2_CO_3_ + 2Al(NO_3_)_3_·9H_2_O = 2Al(OH)_3_ + 6NH_4_NO_3_ + 15H_2_O + 3CO_2_↑3(NH_4_)_2_CO_3_ + 2La(NO_3_)_3_·6H_2_O = 2La(OH)_3_ + 6NH_4_NO_3_ + 9H_2_O + 3CO_2_↑

### Material characterization

Thermogravimetric analysis (TGA) was conducted on the sample precursor and the carbon deposited after the life test (Seiko Instruments EXSTAR TG/DTA 6300) in air with a flow rate of 100 mL min^−1^ and a heating rate of 5 °C min^−1^. The Ni and La contents were determined by X-ray fluorescence (XRF) analysis performed on a 2424XRF instrument (Rh target, X-ray tube maximum power 2.4 kW). X-ray diffraction (XRD, Bruker D-8 Advance diffractometer) was used to examine the phase and crystallinity of the samples over the 2*θ* range from 10 to 80° using a Cu-Kα radiation source (*λ* = 0.154060 nm) with a step scan of 0.02°. The BET surface areas of the samples were determined by N_2_ adsorption–desorption measurements using a Micromeritics ASAP 2020 instrument at 77 K. Prior to analysis, the samples were degassed under vacuum for 30 min at ambient temperature followed by fast-mode degassing at 300 °C for 12 h. The reducibility of the catalysts was examined by H_2_ temperature-programmed reduction (H_2_-TPR) using a continuous-flow tube reactor. For each analysis, 100 mg of catalyst was packed in a quartz tube and a reducing gas containing 9.6% H_2_ in argon flowed into the reactor at a rate of 30 mL min^−1^. The adsorption properties (TPD) of the as-prepared catalysts were determined using a BELCAT-II-T-SP characterization system. CO chemical adsorption was used to determine the dispersion and surface area of metallic Ni in the sample. The chemical composition and elemental state of samples were determined by X-ray photoelectron spectroscopy (XPS, Thermo Scientific ESCALab 250Xi with 200 W monochromatic Al Kα radiation). The obtained binding energies were corrected with reference to C 1s (284.8 eV).

### Catalytic performances

**Fig. 1:**
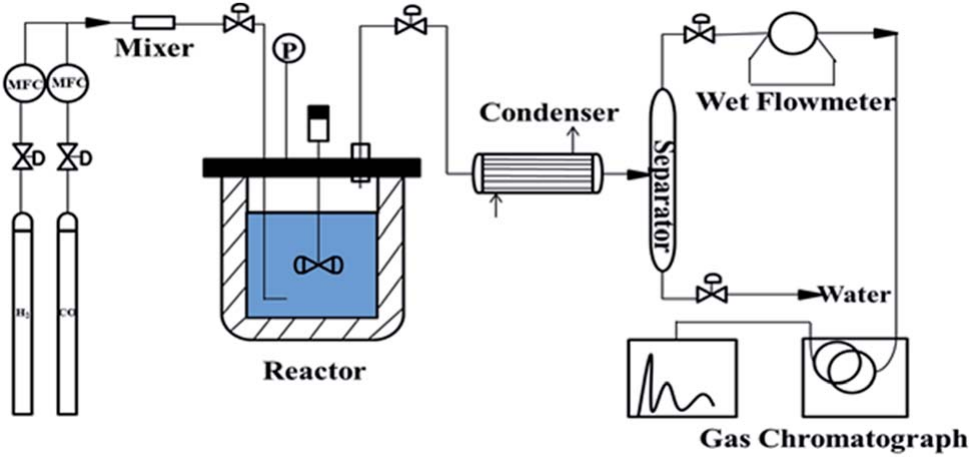
Schematic diagram of the slurry-bed reaction for the syngas methanation reaction.

To determine the catalytic performance, 4.50 g of catalyst and 250 mL liquid paraffin (boiling range of 280–320 °C) were placed in a 1.0 L slurry-bed reactor with a rotating speed of 750 rpm. After the reaction, the mixture gas, including products, was passed through a condenser and were separated in a gas–liquid separator. Water was removed from the bottom of the separator and all of the H_2_, CH_4_, CO_2_, and CO gases in the gas mixture from the top of the separator were quantitatively analyzed by an online gas chromatography system (3000A; Agilent Technologies; TCD) as shown in Fig. 1.

## Results and discussion

### Selection of calcination temperature

**Fig. 2:**
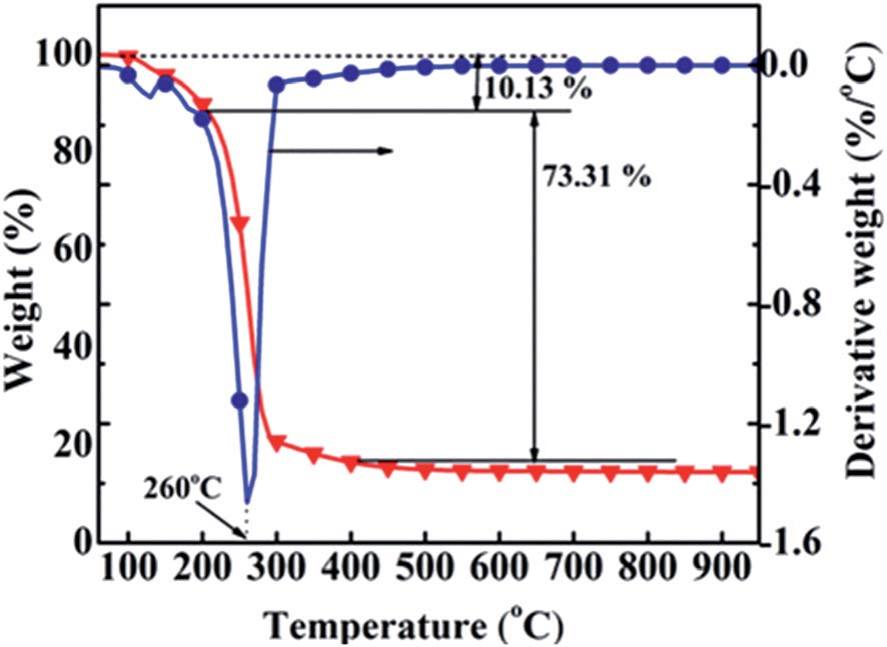
TG/DTG profiles of the precursor.

The calcination temperature range for the precursor was determined by TG/DTG analysis. Fig. 2 gives the TG/DTG profiles of the precursor, indicating that the weight loss process was divided into three phases. The low-temperature phase of 100–200 °C was ascribed to the evaporation of physically adsorbed water with a weight loss of 10.13%. The mid-temperature phase was from 200 to 400 °C with a weight loss of 73.31%. Al(OH)_3_, NiCO_3_, NH_4_NO_3_ and La(OH)_3_ decomposed to Al_2_O_3_ and H_2_O, NiO and CO_2_, NH_3_ and NO_2_, and La_2_O_3_ and H_2_O, respectively, because the decomposition temperatures ranges for NiCO_3_/La(OH)_3_, Al(OH)_3_ and NH_4_NO_3_ were 200–300, about 300 °C and 230 °C,^13,14,24^ respectively. There was almost no weight loss in the high-temperature phase of 400–1000 °C. Thus, 350, 400, 500, 600 and 700 °C were selected as the calcination temperatures, and the corresponding samples were named cat-350, cat-400, cat-500, cat-600, and cat-700, respectively.

### Evaluation of the catalytic performance

The evaluation experiments for the five reduced samples were carried out on the device shown in Fig. 1. Experimental conditions were as follows: space velocity = 3000 mL (g h)^−1^, stirring rate = 750 rpm, temperature = 280 °C, pressure = 1.0 MPa, time = 10 hours, and H_2_/CO = 3.1 : 1 (molar). The main reactions of the methanation process were as follows:

CO methanation reaction

1 CO + 3H_2_ = CH_4_ + H_2_O (Δ*H* = −206 kJ mol^−1^)

Water–gas shift reaction

2 CO + H_2_O = CO_2_ + H_2_ (Δ*H* = −41 kJ mol^−1^)

CO disproportionation reaction

3 2CO = C + CO_2_ (Δ*H* = 17 kJ mol^−1^)

CO_2_ methanation reaction

4 CO_2_ + 4H_2_ = CH_4_ + 2H_2_O (Δ*H* = −165 kJ mol^−1^)

**Fig. 3:**
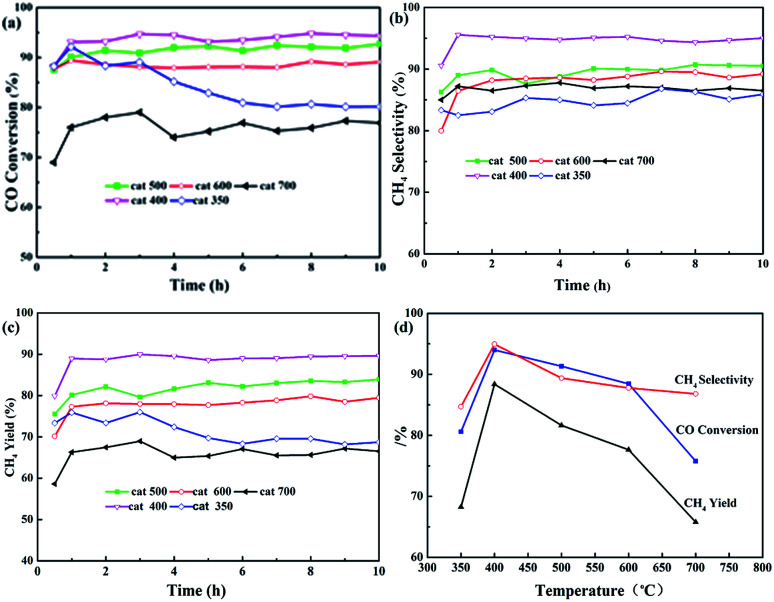
Influence of calcination temperature on CO methanation properties of the samples. (a) CO conversion, (b) CH_4_ selectivity, (c) CH_4_ yield, (d) average of parameters.

Fig. 3 shows the effects of calcination temperature on CO conversion (*X*_CO_), CH_4_ selectivity (*S*_CH_4__), and CH_4_ yield (*Y*_CH_4__). As shown in Fig. 3(a)–(c), the curves of *X*_CO_, *S*_CH_4__, and *Y*_CH_4__ for all the samples may be divided into two stages, namely, the initial phase (about 1 h) and stable phase (9 h, except for cat-350). *X*_CO_ increased by 5 to 10% as shown in Fig. 3(a). For example, in the initial phase, *X*_CO_ increase from 88.0% to 92.1, 94.0, 91.3 and 88.4% based on cat-350, cat-400, cat-500, and cat-600, respectively, and from 68.9% to 75.8% based on cat-700. In the stable phase, *X*_CO_ of cat-400, cat-500, cat-600 and cat-700 was kept at 94.3, 92.0, 89.1 and 73.3%, respectively, and only *X*_CO_ of cat-350 decreased rapidly from 92.1% to about 80% before 6 hours.

In Fig. 3(b), *S*_CH_4__ of cat-400 maintained the best value of 94.9% within 10 h, while that of the others was maintained between 84.7 and 89.6%. In Fig. 3(c), *Y*_CH_4__ of samples is ranked as follows: 88.4% for cat-400 > 81.6% for cat-500 > 77.6% for cat-600 > 68.3% for cat-350 > 65.80% for cat-700. Fig. 3(d) gives the average values of *X*_CO_, *S*_CH_4__ and *Y*_CH_4__ during 10 h for the five samples, in which the curves for catalytic performance show an open downward parabolic tendency with the increase in the calcination temperature. *X*_CO_, *S*_CH_4__ and *Y*_CH_4__ of cat-400 reached the maximum of 94.0, 94.9 and 88.4%, respectively. Therefore, 400 °C was chosen as the optimum calcination temperature.

Table 1 shows the theoretical and experimental contents and loadings of Ni and La in calcined samples. As shown, the Ni and La loadings were in the range of 93.80–95.13% and 91.0–92.5%, respectively, which almost reached the theoretical value.

**Table 1:** XRF results of catalysts^a^

Sample	Ni wt%			La wt%		
	Theor. value	Exper. value	Load	Theor. value	Exper. value	Load
Cat-350	15	14.32	95.47	2	1.85	92.5
Cat-400		14.27	95.13		1.84	92.0
Cat-500		14.16	94.27		1.83	91.5
Cat-600		14.09	93.93		1.82	91.0
Cat-700		14.07	93.80		1.82	91.0

a Theor. value: theoretical value, exper. value: experimental value.

**Fig. 4:**
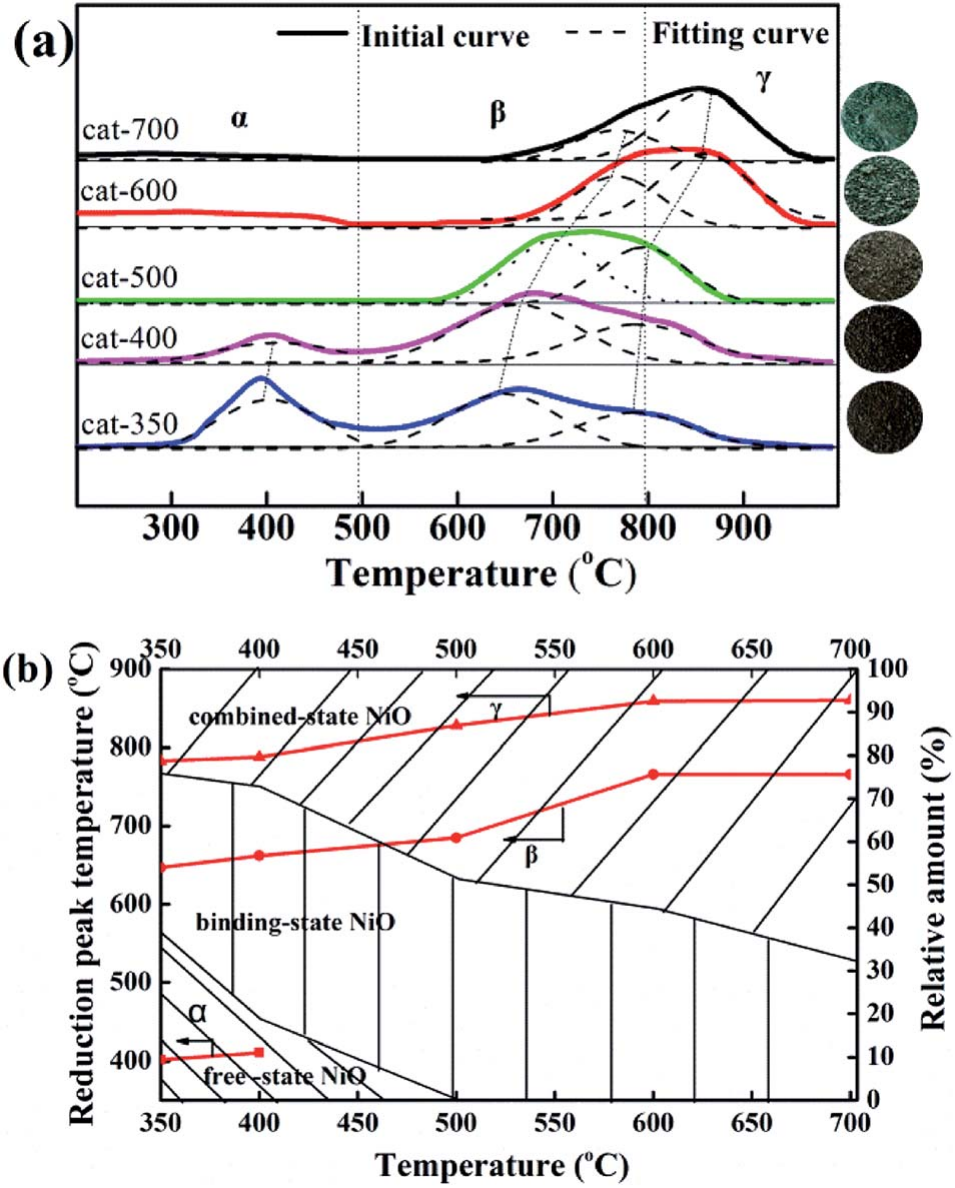
H_2_-TPR profiles (a) and Gaussian fitting analysis (b) for the calcined samples.

As shown in Fig. 4(a), when the calcination temperature increased from 350 to 700 °C, the colors of the samples changed in the sequence of black, grayish-black, gray, gray-green and green, indicating that the calcination temperature deeply affected the types of NiO. According to the peak position of H_2_-TPR profiles, the reducible NiO species could be divided into three types: α-NiO (300–500 °C), β-NiO (500–800 °C), and γ-NiO (800–1000 °C).^25–28^ The α-NiO reduction peak was attributed to the reduction of free NiO, which had almost no interaction with Al_2_O_3_.^24,28^ The β-NiO reduction peak was generated from the reduction of bound NiO and there was a strong interaction between NiO and Al_2_O_3_. The γ-NiO reduction peak corresponds to the reduction of the nickel-aluminum spinel (NiAl_2_O_4_). NiO and Al_2_O_3_ were due to the interactions of NiAl_2_O_4_. Furthermore, there were α-NiO, β-NiO and γ-NiO reduction peaks for both cat-350 and cat-400 samples, whereas the α-NiO reduction peak disappeared, and the peak temperature of β-NiO and γ-NiO increased significantly for other samples. In Fig. 4(b), for the five samples, temperatures of the three reduction peaks increased, that is, the temperatures of the α-peak, the β-peak and the γ-NiO increased from 402 to 411 °C, 647 to 766 °C, and 783 to 860 °C, respectively. Thus, the calcination temperature influenced the types of NiO present. Secondly, the relative content of α-NiO decreased from 39.0% to 0 when the calcination temperature increased from 350 to 500 °C, while the relative content of the β-type NiO increased from 24.1% to 67.8% as the calcination temperature increased from 350 to 700 °C. The β-NiO content of cat-400 appeared as a parabolic curve with a downward opening, which reached the maximum at 54%. As such, 400 °C was the optimum calcination temperature. Thirdly, for cat-400, α-NiO, β-NiO and γ-NiO were present and their relative contents were 18.8%, 54.0% and 27.2%, respectively. For cat-500, cat-600 and cat-700, there was only β-NiO and γ-NiO.

In summary, calcination temperature affects the types of NiO present in the samples. The reason comes from the following two aspects. The first aspect is the three existing types of NiO. The influence degree order is bound NiO ≫ free NiO > combined NiO, which is consistent with literature.^27–30^ The second is the relative content ratio of the three existing types and a reasonable ratio is 1 : 3 : 2.

**Fig. 5:**
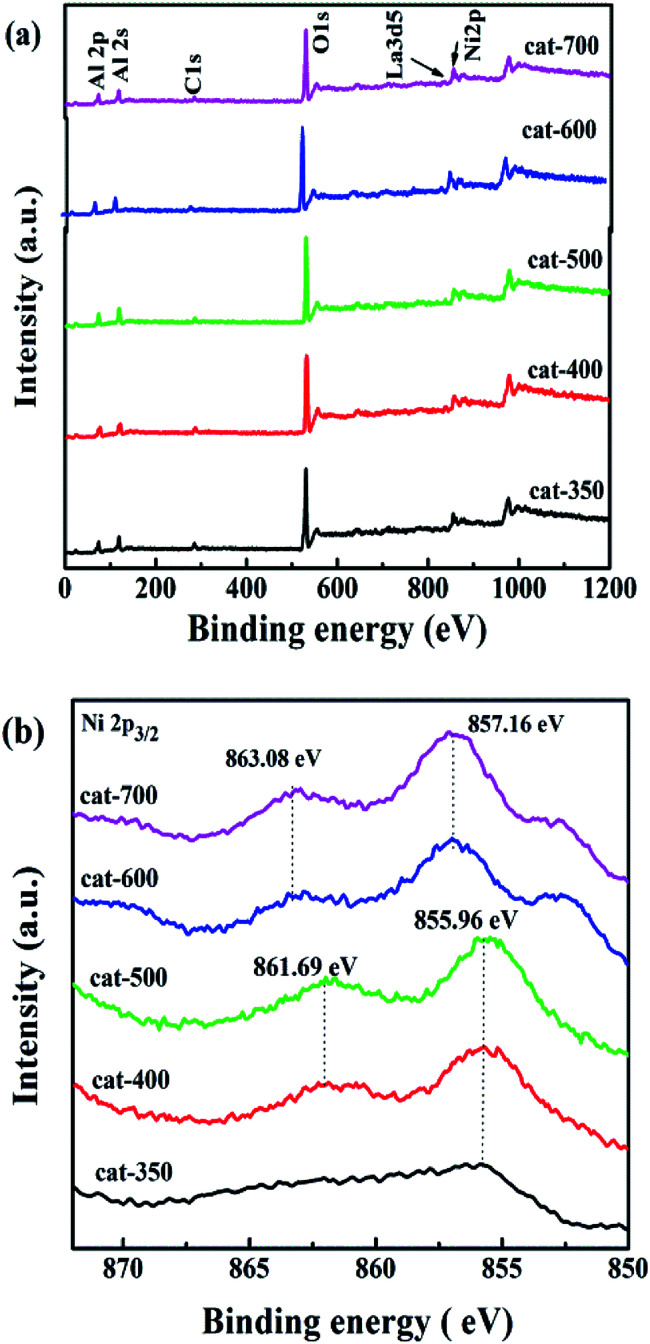
Survey XPS spectra (a) and Ni 2p spectra wide scans (b) for all samples.

Fig. 5 shows the XPS spectra of all samples. According to Fig. 5(a), O, La, Ni and Al species on the surface of the samples show similar O 1s, La 3d5, Ni 2p and both Al 2s and Al 2p peaks, respectively. According to Fig. 5(b), the position of the Ni 2p_3/2_ peak is in the range of 855.96–863.08 eV, indicating that there are two bands, where the first one located at 855.96–857.16 eV is associated with NiO exhibiting weak interactions between NiO and Al_2_O_3_,^28^ and the second one located at 861.69–863.08 eV is related to Ni^2+^ in the formation of NiAl_2_O_4_ with strong interaction between NiO and Al_2_O_3_. The binding energy values of Ni 2p_3/2_ for cat-350, cat-400, and cat-500 were at 855.96 and 861.69 eV, which were lower than those of 857.16 and 863.08 eV for cat-600 and cat-700. Interestingly, this observation suggests the existence of strong interactions between NiO and Al_2_O_3_ of NiAl_2_O_4_ while the calcination temperature changed from 350 °C to 700 °C. ^31,32^ This result is in agreement with that of H_2_-TPR.

**Fig. 6:**
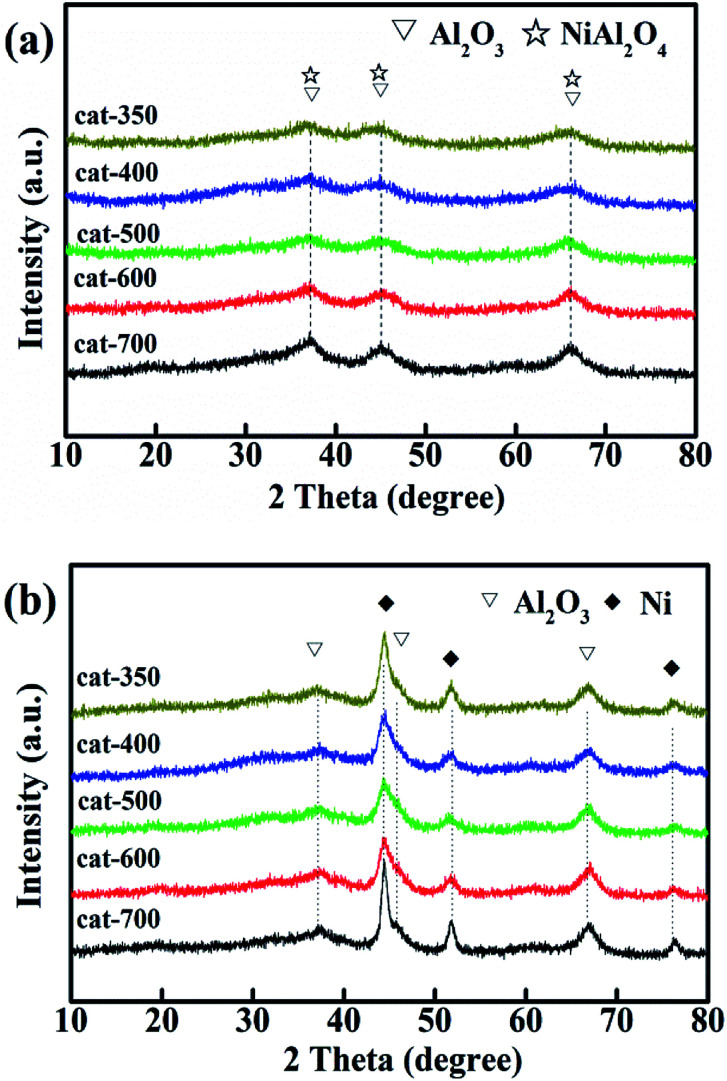
XRD patterns of calcined (a) and reduced samples (b).

Fig. 6 shows the XRD patterns of calcined (a) and reduced samples (b). As shown in Fig. 6(a), for the calcined samples, the characteristic diffraction peaks at 2*θ* = 37.5°, 45.5° and 66.4° are attributed to γ-Al_2_O_3_ (JPCDS 10-0425) and NiAl_2_O_4_ (JPCDS 10-0339). The diffraction peaks became stronger and sharper as the calcination temperature increased. This is consistent with the H_2_-TPR results that when the calcination temperature increased from 350 °C to 700 °C, the relative content of NiAl_2_O_4_ was also enhanced from 24.1 to 67.8% because of the transition of NiO from the free-type to bound-type to combined type. However, the characteristic diffraction peaks of La_2_O_3_ and NiO (JPCDS 73-1519) at 2*θ* = 43.3°, 62.8° and 75.4° did not appear, which means that the NiO and La_2_O_3_ species were highly dispersed.^33–35^

Furthermore, according to Fig. 6(b), there were new characteristic diffraction peaks attributed to Ni (JPCDS 04-0850) at 2*θ* = 44.5°, 51.8° and 76.4° for all samples reduced at 800 °C for 3 h, indicating that NiAl_2_O_4_ was reduced to Ni, and the Ni crystallites were well dispersed on Al_2_O_3_.^6,31,33–36^

Fig. S1† shows the effect of calcination temperature on the Ni grain size of the reduced samples according to Scherrer's formula (*D* = 0.89*λ*/(*β* cos *θ*)) based on the peak of Ni at 2*θ* = 51.8° in Fig. 6(b). Fig. S1† shows that the Ni grain size of cat-400 reached a minimum (7.08 nm), and those of cat-500 and cat-600 were less than 8.0 nm, whereas those of the remainder were more than 9.0 nm. Thus, combined with the results of H_2_-TPR, it has been shown that the types of NiO can significantly affect the Ni grain size. This is why cat-400 showed the maximum values of *X*_CO_, *S*_CH_4__, and *Y*_CH_4__ in the 10 h evaluation experiments.^25,27^

**Fig. 7:**
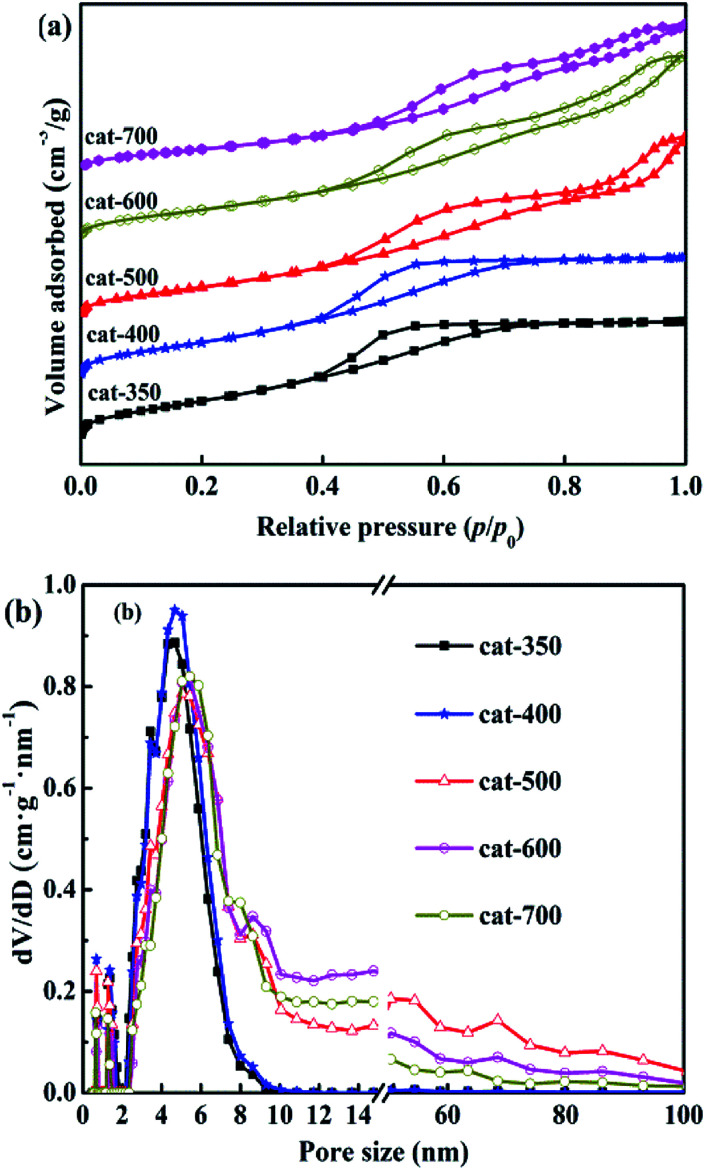
N_2_ absorption–desorption isotherms (a) and pore size distributions (b) of samples.

N_2_ adsorption–desorption isotherms and pore size distributions of the calcined samples are shown in Fig. 7(a) and (b). According to the IUPAC classification method, all samples exhibited type IV isotherms with hysteresis loops under relative pressure of (*p*/*p*_0_) ≤ 0.4–0.45. As shown in Fig. 7(a), when the relative pressure of *p*/*p*_0_ was more than 0.4–0.45, the N_2_ adsorption amount increased rapidly due to capillary condensation, causing hysteresis loops to appear,^33–35^ indicating the presence of mesoporous structures in the five samples. However, there were significant differences in the hysteresis loop shapes of the five samples. Cat-350 and cat-400 showed type H2 isotherms, indicating that they were agglomerated with uniform size and narrow pore-size distribution, and the *p*/*p*_0_ range was 0.4–0.7. The maximum adsorption amount was due to saturation adsorption, and the pore size distributions were almost mesopores (2–9 nm). Cat-500, cat-600 and cat-700 all showed obvious H3 type hysteresis loops, which mean that the pore structures were slits formed by the stacking of tabular particles, and the *p*/*p*_0_ range was 0.4–1.0 without saturated adsorption capacity.

In Fig. 7(b), it can be seen that the pore-size distribution of the three samples was between 2 and 100 nm. Furthermore, the initial relative pressure of the hysteresis loops gradually increased from 0.4 to 0.45 with the increase in the calcination temperature. The relative pressure of capillary condensation occurring in the samples' mesoporous holes gradually increased, indicating that the mesoporous diameters of the samples gradually increased.^28,36^ In summary, the calcination temperature has significant effects on the characteristics of the pore structures in the samples, and when the calcination temperature was 350–400 °C, the pore size was distributed within the mesopores.

**Fig. 8:**
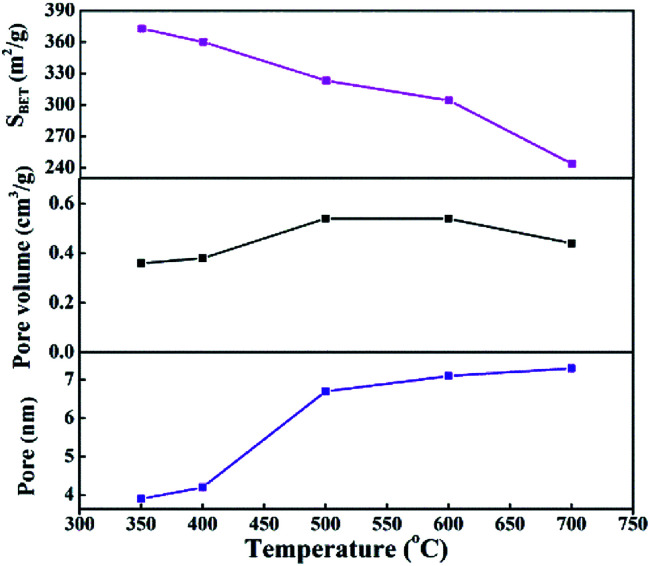
The influence of calcination temperature on the pore structure of the calcined samples.

As shown in Fig. S2,† the effects of calcination temperature on the dispersion, surface area and particle diameter of Ni were investigated through CO chemisorption testing of the reduced samples. The Ni dispersion and surface area of cat-400 were 11.3% and 10.73 m^2^ g_cat_^−1^, respectively. The dispersion and surface area of Ni of cat-700 were only 7.2% and 5.97 m^2^ g_cat_^−1^, and those of cat-350 were 8.7% and 8.46 m^2^ g_cat_^−1^, respectively, which coincided with the results shown in Fig. S1.† Obviously, the larger dispersion and surface area of Ni can provide more active sites, and show better catalytic performance. The sizes of Ni crystallites in cat-350 were large but the dispersion degree was low. The size of Ni grains of cat-400 were the smallest (8.77 nm),^28^ while those of cat-700 and cat-350 were 12.54 nm and 11.24 nm, respectively. Compared to the size calculated by Scherrer's formula, the difference between the two results was less than 15%. Thus, the analysis results in Fig. S1 and S2† are reliable. The pore size distribution is in the range of 3.9−7.3 nm as exhibited in Fig. 8, indicating the presence of mesoporous structures in all catalysts. The BET specific surface area of the catalysts decreases from 372.8 m^2^ g^−1^ to 243.6 m^2^ g^−1^ with the increased temperature as listed in Fig. 8.

**Fig. 9:**
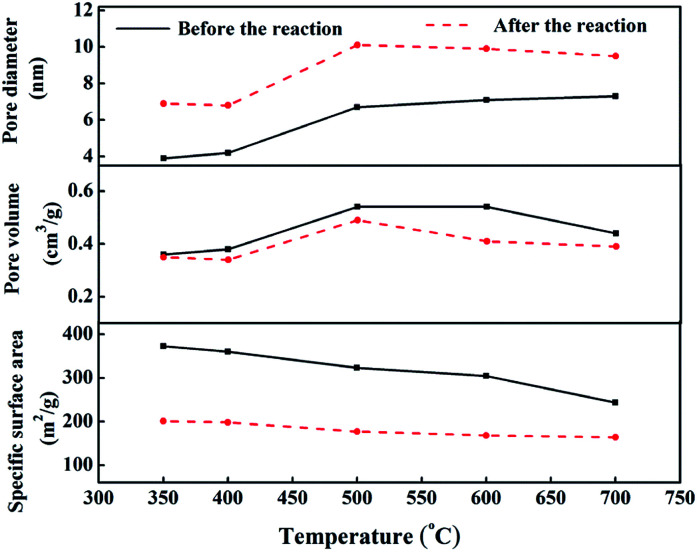
Comparison of pore structure parameters before and after the reaction.

Fig. 9 shows the pore structure parameters before and after the CO methanation reaction. The average pore diameter of the five samples increased by 2–3 nm after the reaction and the specific was surface area reduced to 100–200 m^2^ g^−1^ after the reaction. This was mainly due to the collision between particles while the catalyst was scoured with the rapid liquid paraffin flow in the slurry-bed reactor. The formation of carbon deposits from the CO disproportionation, shown in eqn (3), may make these problems more serious. However, compared to the 10 h evaluation results, the catalytic activities of all samples did not exhibit a declining trend, except for cat-350. This means that in terms of the reaction, the average pore diameter of the catalysts changed within the nanoscale, and the change in the specific surface area was within a certain range, which had little effect on its activity, indicating that the pore structure parameters of the samples were not the sensitive factors affecting the activity.^37^

**Fig. 10:**
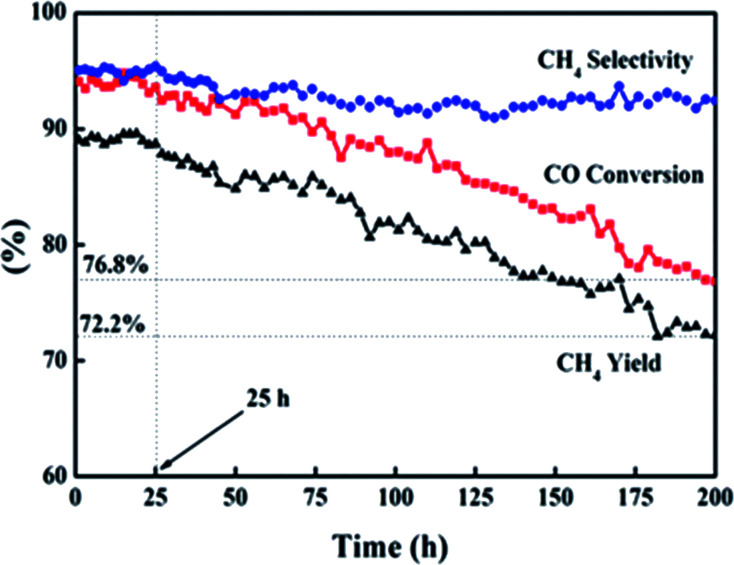
Cat-400 with time in the lifetime test.

The lifetime of a catalyst is a key consideration in the economical production of syngas methanation. Deposited carbon, an intermediate product during methanation, can lead to the deactivation of the catalyst.^38–41^Fig. 10 exhibits the catalytic performance of cat-400 at 238 °C and 0.2 MPa within 200 h. The catalytic activity was stable within the first 25 h, that is, *X*_CO_, *Y*_CH_4__, and *S*_CH_4__ remained at 94.0, 89.1, and 94.9%, respectively. These values are consistent with the results of the evaluation experiment over 10 h. Afterwards, within 175 h, *X*_CO_ and *Y*_CH_4__ dropped to 76.8% and 72.2% with the decreasing rates of 0.1 and 0.11% per h, respectively, and *S*_CH_4__ was basically stable at 93%.

**Fig. 11:**
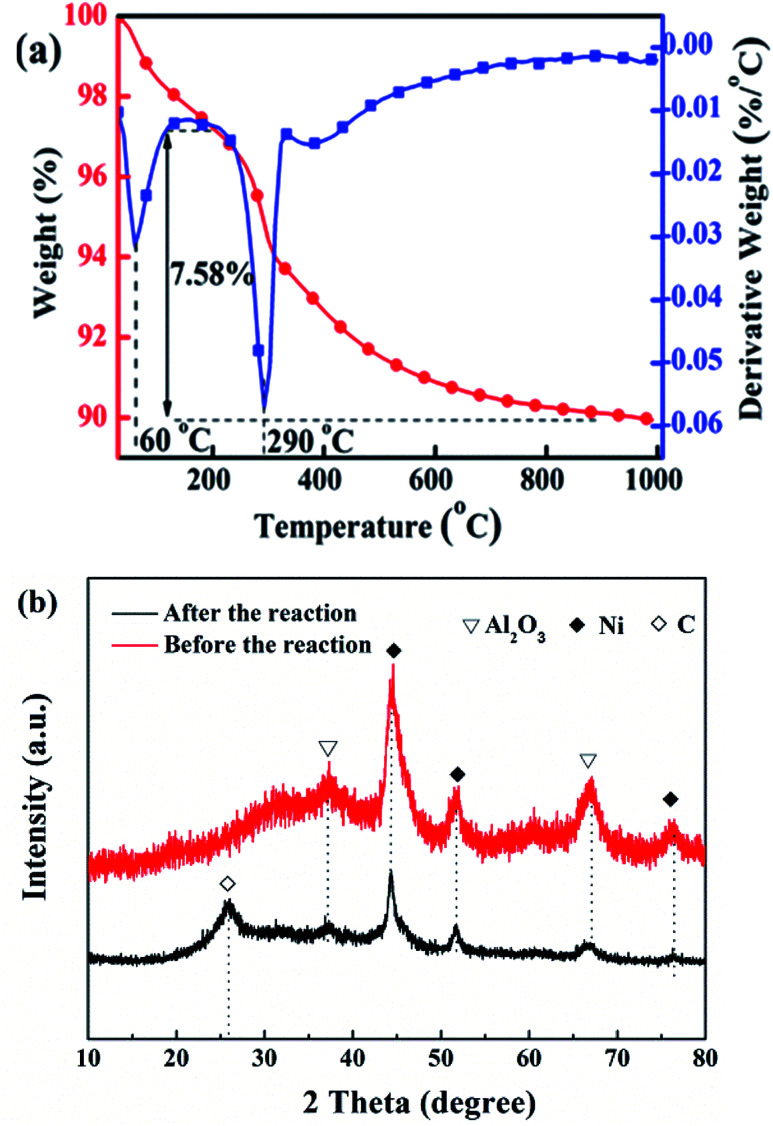
TG-DTG (a) and XRD patterns (b) of cat-400 after lifetime testing.

Fig. 11 shows the TG-DTG curves (a) and XRD patterns (b) of cat-400 after a lifetime test of 200 h. There are two peaks for the loss of weight in Fig. 11(a). The temperature of the first one was about 100 °C, corresponding to the weight loss of water, and the temperature of the second was 290 °C due to the oxidative weight loss of amorphous carbon.^28,31,38–41^ This indicates that carbon deposition occurs during the 200 h reaction because of the CO disproportionation during the 200 h reaction. The amorphous carbon types mainly formed because the reaction temperature was low, only about 280 °C. The weight loss at 200–1000 °C, *i.e.*, the carbon deposition quantity, was 7.58%, and the carbon deposition rate was about 0.38 mg (h g_cat_)^−1^. Fig. 11(b) shows the XRD patterns of cat-400 before and after the lifetime test. After the stability evaluation test, there was no change in the intensity of the Al_2_O_3_ diffraction peak in cat-400. However, the intensity of the Ni diffraction peak was weakened and diffused as compared with the fresh cat-400. Combining these results from the TG-DTG analysis, we conclude that the decrease in the catalytic activity was not due to Ni sintering but was caused by the amorphous carbon from the CO disproportionation reaction. There is no carbon diffraction peak in Fig. 11(b), which further confirms the surface coating by amorphous carbon.

**Fig. 12:**
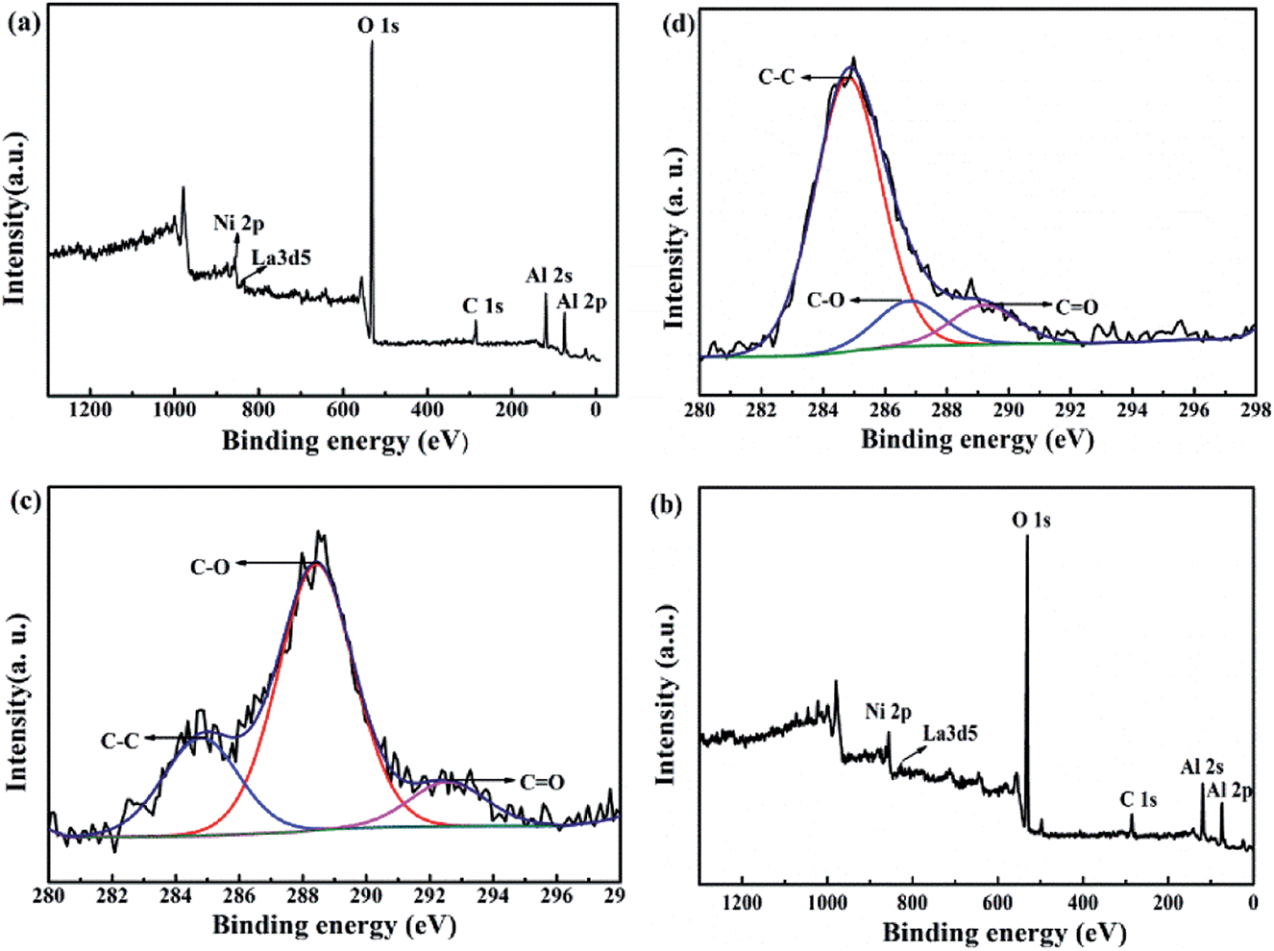
Comparison of survey XPS spectra before (a) and after (b) a lifetime test of 200 h and C 1s spectrum-wide scans of reduced cat-400 before (c) and after (d) the lifetime test.

Fig. 12 shows the XPS survey spectra before (a) and after (b) the lifetime test over 200 h. The C 1s spectrum-wide scans of cat-400 before (c) and after (d) the lifetime test for 200 h are also shown. On comparing Fig. 12(a) and (b), it can be seen that the peak location of each element near the surface of cat-400 was almost the same as the binding energy: 853.0 for Ni 2p, 835.6 eV for La 3 d5, 531.2 eV for O 1s, 288.4 eV (C–O), 284.8 eV (C–C) and 292.6 eV (C=O) for C 1s,^38–41^ and 74.38 eV for Al 2p. This indicates that Ni, La, O, C and Al were still near the surface of cat-400 after the test.

There were three kinds of combined carbon species near the surface of the reduced cat-400 sample as shown in Fig. 12(c) and (d) after the stability evaluation experiment. The (285 ± 0.3) eV peak corresponds to the amorphous carbon sp^3^ hybridization, the (288 ± 0.3) eV peak was assigned to a C–O single bond, and peaks at energies higher than 289 eV were ascribed to the double bond of carbon–oxygen. Fitting (c) and (d) provided C 1s peak data for cat-400 before and after the lifetime test of 200 h, shown in Table 2.^42–45^

**Table 2:** C 1s peak fitting XPS data for reduced cat-400 before and after the lifetime test of 200 h

Sample	Carbonous type	Binding energy (eV)	*n*_c_ (%)
Fresh	C 1s C–O	288.4	65.2
	C 1s C–C	284.8	23.9
	C 1s C=O	292.6	10.9
Spent	C 1s C–O	286.8	12.7
	C 1s C–C	284.8	76.3
	C 1s C=O	289.2	11.0

Table 2 shows the C 1s peak fitting XPS data for the reduced cat-400, before and after the lifetime test of 200 h. C–O is the main carbonous type, and the C–O content in the sample was 65.2% of the total carbon content before the lifetime test.^40,46^ After the stabilization experiment, the near-surface of the sample was mainly composed of carbon–carbon single bonds in the form of amorphous carbon, which accounted for 76.3% of the total carbon content. This was mainly due to the CO disproportionation reaction in eqn (3) when the methanation reaction was extended to 200 h.^47–49^ In combination with XRD and TG-DTG analysis, inactive amorphous carbon was formed and attached to the surface of cat-400 after the stability test, resulting in decreased catalytic activity. The XPS results further confirmed that the surface was covered by amorphous carbon after the 200 h reaction.

## Conclusions

The effects of calcination temperature on the structure–activity relationship of the Ni–La/Al_2_O_3_ catalyst prepared by the mechanochemical method for the syngas methanation reaction were comprehensively investigated. The interactions between NiO and Al_2_O_3_ support increased when the temperature increased from 350 °C to 700 °C because the relative content of free-state NiO decreased, whereas the relative contents of the bound and combined states of NiO increased. Cat-400 showed the highest catalytic activity, with the specific surface area of 372.8 m^2^ g^−1^ and the smallest (7.08 nm) Ni^0^ crystalline grain size, which reduced the internal diffusion resistance and offered more reactive sites. Cat-400 exhibited good stability and catalytic activity in the lifetime test of 200 h. From the TG-DTG, XRD and XPS analyses, the decrease in the catalytic activity of cat-400 resulted mainly from the deposition of amorphous carbon on the catalyst surface *via* CO disproportionation. This work not only shows the importance of calcination temperature, which can determine the physicochemical properties of catalyst and the catalytic performance on methanation of syngas, but it can also be used as a reference for material synthesis for other reactions.

## Conflicts of interest

There is no conflicts to declare.

## Supplementary Material

RA-010-C9RA09674D-s001
