# Supplementary material for: Effects of calcination temperatures on the structure–activity relationship of Ni–La/Al2O3 catalysts for syngas methanation
Source: RSC Adv. 2020 Jan 27;10(7):4166–74. doi: 10.1039/c9ra09674d (PMC9048969; doi:10.1039/c9ra09674d)
Supplement: RA-010-C9RA09674D-s001 [file RA-010-C9RA09674D-s001.pdf]

Electronic Supplementary Material (ESI) for RSC Advances.
This journal is © The Royal Society of Chemistry 2020

Supporting information file for

Effects of calcination temperature on structure-activity of Ni-La/Al2O3 catalyst for syngas methanation

Hongli Wu,[a,b] Meng Zou,[a] Lisheng Guo,[b] Fengyun Ma,*[a] Wenlong Mo,[a] Yuming Yu,[a] Inamullah Mian[a] and Noritatsu Tsubaki*[b]

[a] *Key Laboratory of Coal Cleaning Conversion and Chemical Engineering Process Xinjiang Uyghur Autonomous Region, College of Chemistry and Chemical Engineering, Xinjiang University, Urumqi, Xinjiang 830046.*

[b] *Department of Applied Chemistry, School of Engineering, University of Toyama, Gofuku 3190, Toyama 930-8555, Japan.*

Fengyun Ma: ma_fy@126.com

Noritatsu Tsubaki: tsubaki@eng.u-toyama.ac.jp

Fig. S1 Effect of calcination temperature on Ni grain size for reduced sample

Fig. S2 Effects of calcination temperature on dispersion, S_{BET} and diameter of Ni.

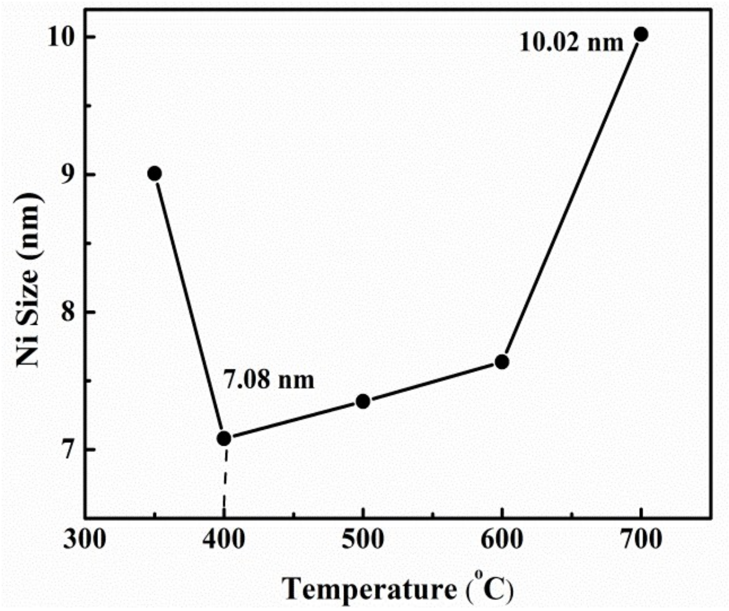

Fig. S1 Effect of calcination temperature on Ni grain size for reduced samples

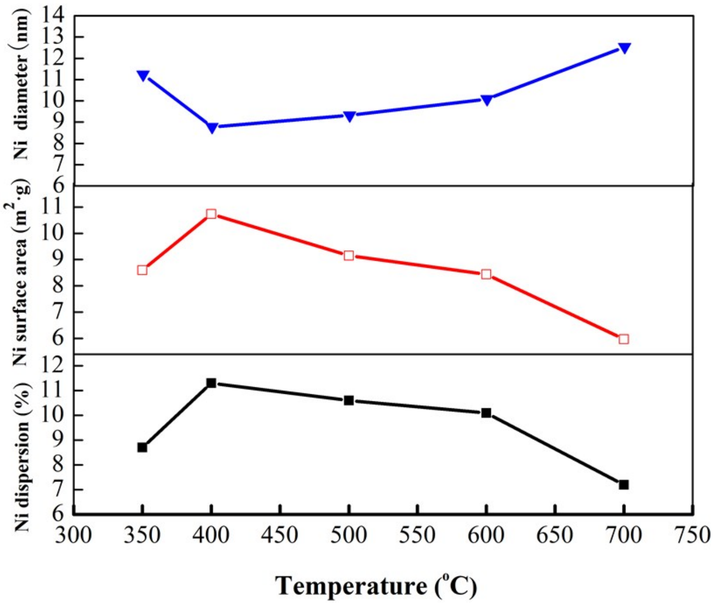

Fig. S2 Effects of calcination temperature on dispersion, S_{BET} and diameter of Ni.
